# CMOS-MEMS Z-Axis Magnetic Field Sensor with an Additional Collector

**DOI:** 10.3390/mi17091029

**Published:** 2026-08-29

**Authors:** Zhi-Xuan Dai, Rong-Wei Tsai, Qing-Hua Shih, Cheng-Chih Hsu

**Affiliations:** 1 Department of Bio-Industrial Mechatronics Engineering, National Chung Hsing University, Taichung 402, Taiwan; zxdai@nchu.edu.tw (Z.-X.D.); b10944050@gmail.com (Q.-H.S.); 2 ETC2, Taiwan Semiconductor Manufacturing Company, Tainan 741, Taiwan; welly21542035@gmail.com; 3 Department of Electro-Optical Engineering, National United University, Miaoli 360, Taiwan

**Keywords:** CMOS-MEMS, magnetic field sensor, magnetotransistor, post-CMOS process, shallow trench isolation

## Abstract

A complementary metal oxide semiconductor (CMOS)-compatible *z*-axis magnetic field sensor incorporating an additional collector is proposed to enhance magnetic sensing performance. The sensor consists of four identical magnetic sensing elements arranged in a cross-shaped configuration, while shallow trench isolation (STI) and a post-CMOS cavity structure are employed to suppress substrate leakage current and improve electrical isolation. The sensing characteristics were investigated using three-dimensional TCAD simulations to analyze carrier transport and current density distributions under different magnetic fields. The simulation results confirmed that the structure effectively enhances the differential output response and magnetic sensitivity. The device was fabricated using a commercial CMOS process followed by a simple post-CMOS micromachining process. Optical microscope and scanning electron microscope observations verified the successful formation of the sensing structure and the cavity beneath the sensing elements. The sensor was experimentally characterized under magnetic fields ranging from −300 to 300 mT. The measured results exhibited excellent linearity over the entire measurement range. The sensor achieved a measured sensitivity of 120 mV/T. Owing to its high sensitivity, simple fabrication process, and full compatibility with standard CMOS technology, the magnetic field sensor is promising for integrated microsystems, industrial monitoring, and intelligent sensing applications.

## 1. Introduction

Magnetic field sensors (MFSs) have attracted considerable attention because they enable non-contact detection, high reliability, fast response, and low power consumption [1,2,3]. These advantages make them indispensable components in modern electronic systems for magnetic field measurement, position detection, current monitoring, and motion control [4,5]. With the rapid development of complementary metal oxide semiconductor (CMOS) technology and microsystem integration, CMOS-compatible MFSs have become increasingly attractive owing to their low fabrication cost, compact size, and capability for monolithic integration with signal-processing circuits [6,7,8,9]. Consequently, MFSs have been widely applied in numerous fields. Typical applications include position and displacement sensing in industrial automation [10], current sensing and power management in electrical systems [11], speed and rotational angle detection in automotive electronics [12], electronic compasses and navigation systems in consumer electronics [13], and biomedical instruments for magnetic field measurement and magnetic particle detection [14]. Owing to the continuously increasing demand for high sensitivity, miniaturization, and CMOS compatibility, the development of high-performance CMOS-based MFSs remains an important research topic for integrated microsystems and intelligent sensing applications.

Microelectromechanical systems (MEMS) technology has been widely applied to fabricate microsensors [15,16,17,18], microactuators [19,20,21,22], and micro-components [23,24,25,26]. MEMS-based MFSs offer several advantages, including miniaturization, low power consumption, batch fabrication, and low manufacturing cost. Recently, considerable efforts have been devoted to the development of MEMS MFSs. Dai et al. [9] proposed a *z*-axis MFS based on a CMOS magnetotransistor fabricated using a standard CMOS process without MEMS post-processing. The device employed silicon with n-well, n+ emitter, n+ collector, and p+ base regions and achieved a measured sensitivity of 19.2 mV/T, although its *z*-axis sensitivity was relatively low. Chen et al. [27] developed a three-axis MFS using a commercial CMOS process. The sensor integrated x/*y*-axis magnetic field-effect transistors (MAGFETs) and a *z*-axis MAGFET with shallow trench isolation (STI) and polysilicon gates, achieving a measured *z*-axis sensitivity of 27.8 mV/T. Although the device exhibited low cross-sensitivity and full CMOS compatibility without post-processing, its *z*-axis sensitivity remained limited. Wu et al. [28] reported a CMOS-compatible MFS incorporating x/y- and *z*-axis magnetotransistor sensing elements with STI isolation. The sensor achieved a measured *z*-axis sensitivity of 119 mV/T. Zhao et al. [29] fabricated a monolithically integrated three-axis MFS on a high-resistivity p-type silicon wafer using MEMS technology. The device integrated four silicon magnetic-sensitive transistors and one Hall sensor on a single chip and achieved a measured *z*-axis sensitivity of 77.4 mV/T. However, its fabrication process required precise structural alignment and multiple ion-implantation steps. Sileo et al. [30] developed a MEMS-based three-axis Hall MFS consisting of one planar Hall sensor and two out-of-plane Hall sensors fabricated on a GaAs heterostructure. The sensing structure employed an AlGaAs/InGaAs/GaAs two-dimensional electron gas multilayer with a strained InGaAs/GaAs bilayer and achieved a *z*-axis sensitivity of 30 mV/T. However, the use of III–V semiconductor materials, free-standing suspended structures, and complex micromachining significantly increased the fabrication complexity and cost. Therefore, the development of a CMOS-compatible MFS with high *z*-axis sensitivity, a simple fabrication process, and low manufacturing cost remains an important research challenge.

In this study, a CMOS-MEMS micro magnetic field sensor (μMFS) is proposed, fabricated, and characterized using a commercial CMOS process followed by a simple post-CMOS micromachining process. The sensor incorporates an additional collector to enhance carrier transport and improve magnetic sensitivity. Furthermore, four identical sensing elements are arranged in a cross-shaped configuration to increase the output signal and improve measurement stability. The combination of STI and post-CMOS cavities effectively reduces substrate leakage current and enhances electrical isolation. The experimental results demonstrate that the design achieves high sensitivity while maintaining full compatibility with standard CMOS technology. Compared with our previously reported CMOS-MEMS magnetic field sensor with isolated cavities [9], the present device introduces an additional collector into the magnetotransistor structure. The additional collector enhances the electric field distribution and improves carrier transport toward the sensing collector, resulting in a higher output response and improved *z*-axis magnetic sensitivity while maintaining the same CMOS-MEMS fabrication process.

## 2. Design of the Micro Magnetic Field Sensor

**Figure 1:**
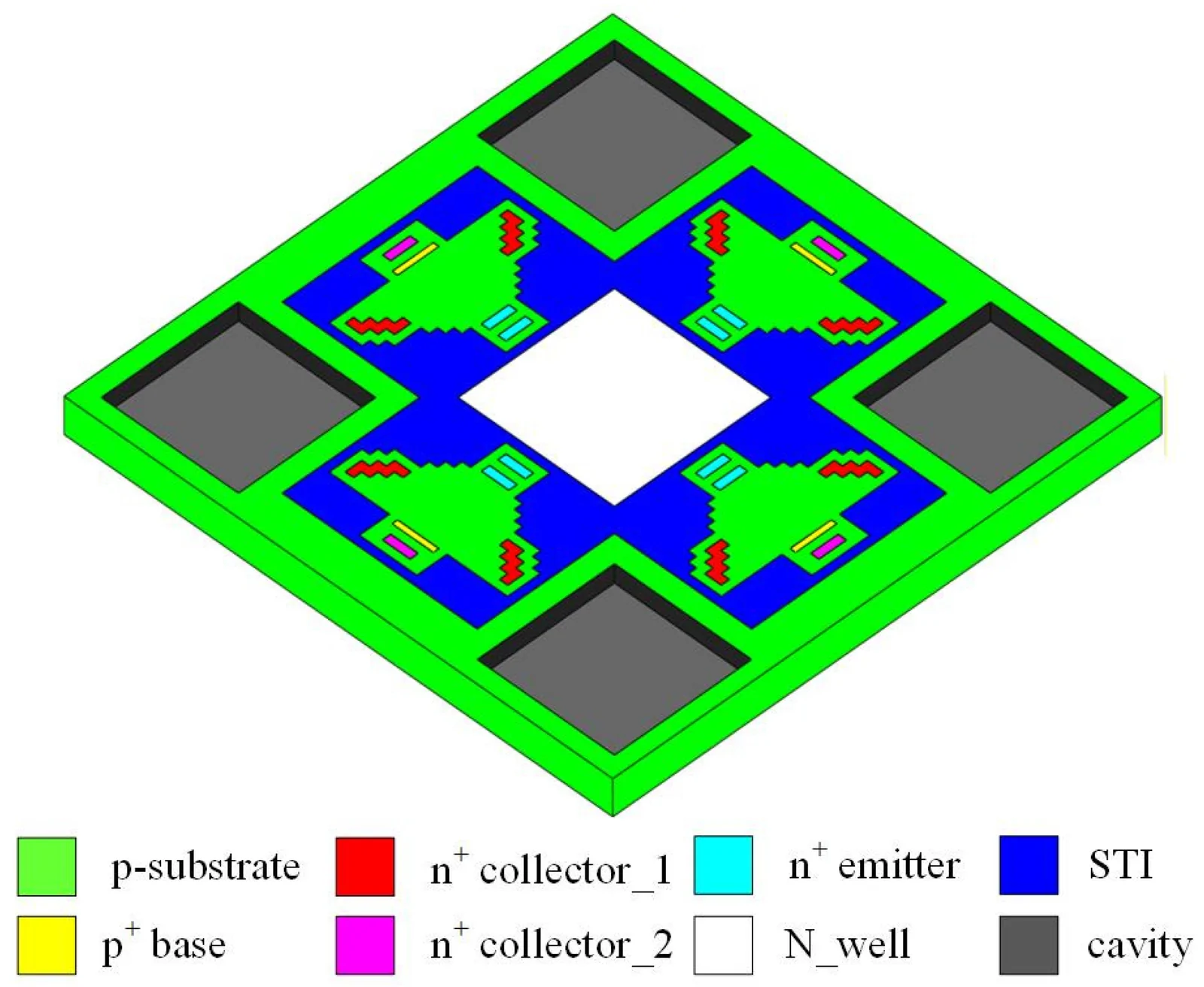
Structure of the micro magnetic field sensor (μMFS).

**Figure 2:**
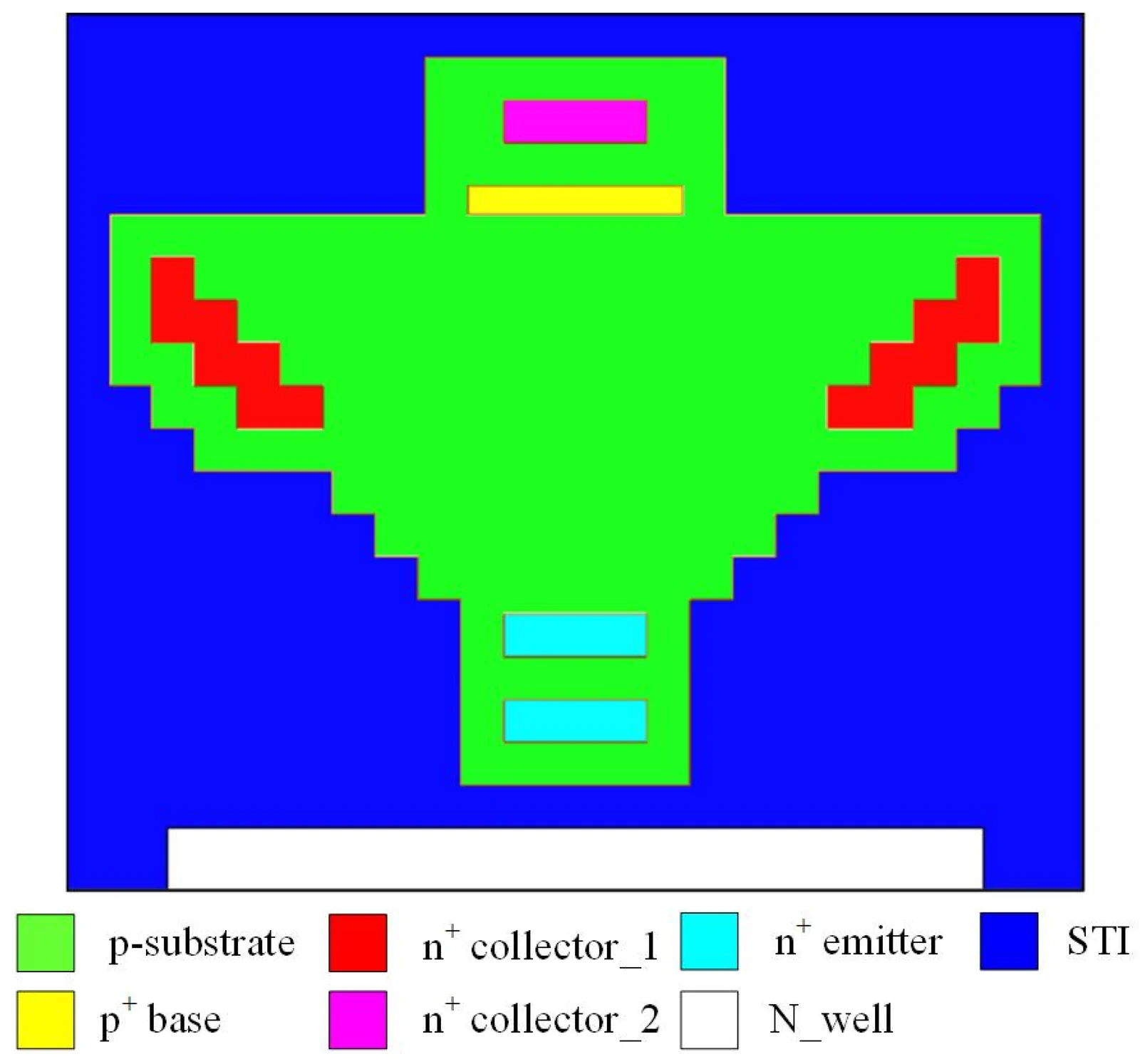
Structure of a magnetic field sensing element.

Figure 1 illustrates the overall architecture of the *z*-axis μMFS, which consists of four identical magnetic sensing elements arranged in a cross-shaped configuration. Figure 2 shows the detailed structure of a single magnetic sensing element. The outputs of the four sensing elements are combined to increase the total sensing current. The generated current difference is then converted into an output voltage by external resistors, providing a differential voltage signal for magnetic field measurement. Each sensing element is fabricated on a p-type silicon substrate and consists of one emitter, one base, two primary collectors (Collector_1), and one additional collector (Collector_2), as shown in Figure 2. A distinctive feature of the design is the incorporation of an additional collector together with an additional emitter, which introduces an auxiliary parasitic negative-positive-negative (NPN) transistor within the magnetic sensing element. The additional collector strengthens the internal electric field distribution and improves the guidance of charge carriers toward the collecting regions, whereas the additional emitter increases carrier injection into the active region. These two structural modifications effectively enhance carrier transport efficiency and increase the current difference generated under an external magnetic field, leading to improved output voltage and magnetic sensitivity.

The base electrode is intentionally designed with a relatively narrow width compared with the other electrodes. In a bipolar junction transistor, a narrow base region shortens the carrier transport path and significantly reduces carrier recombination within the base. Consequently, a greater number of electrons can reach the collector region, resulting in higher current gain and enhanced transistor amplification. This design also contributes to improving the magnetic response because the Lorentz-force-induced carrier deflection can be converted into a larger differential collector current.

Electrical isolation is another important feature of the sensor. The sensing element is surrounded by STI oxide automatically generated during the standard CMOS layout design. Together with the depletion region formed by the N-well structure, the STI effectively suppresses substrate leakage current and isolates the active device from surrounding electrical noise. Furthermore, after completion of the standard CMOS process, the silicon substrate surrounding each sensing element is selectively removed through a post-CMOS micromachining process to form isolated cavities. The combination of STI isolation, N-well depletion barriers, and air cavities substantially reduces parasitic current leakage through the silicon substrate and minimizes substrate coupling effects. This multiple-isolation strategy significantly improves the electrical characteristics of the magnetic sensor, enabling higher sensitivity and more stable operation.

The electrical characteristics of the μMFS were investigated using Synopsys Sentaurus TCAD to verify the sensing mechanism and evaluate the influence of the structural design on device performance. Unlike circuit-level simulations, TCAD provides a physics-based numerical solution that simultaneously considers semiconductor transport phenomena, electrostatic field distribution, carrier recombination, and magnetic field coupling. Therefore, it enables a comprehensive analysis of carrier behavior inside the magnetic sensing element under various operating conditions. The three-dimensional simulation model was established according to the actual device geometry and doping profiles employed in the CMOS fabrication process. The p-type silicon substrate, N-well, emitter, base, and collector regions were incorporated into the simulation model to accurately reproduce the physical structure of the sensor. A refined mesh was generated in the active regions and at the p–n junctions to improve numerical accuracy, while a relatively coarser mesh was adopted in electrically inactive regions to reduce computational time. Appropriate electrical contacts were assigned to the emitter, base, collectors, and additional collectors, and the corresponding bias voltages were applied as the electrical boundary conditions. The simulation was performed at a temperature of 300 K. The three-dimensional device geometry and doping profiles were established according to the layout and fabrication process of the sensor. A uniform magnetic field perpendicular to the device surface (*z*-axis) was then introduced to evaluate the magnetic response of the sensor. Under the applied bias conditions, the carrier transport inside the magnetic transistor was calculated by solving the coupled semiconductor transport equations, including Poisson’s equation together with the electron and hole continuity equations. The interaction between the flowing carriers and the external magnetic field generates the Lorentz force, which deflects the carrier trajectories toward one side of the sensing element. This carrier redistribution modifies the current collected by the two primary collectors and produces a differential output signal. The simulated current density distribution was subsequently analyzed to visualize the carrier transport path and the magnetic-field-induced current deflection. Based on the calculated collector currents, the output voltage of the magnetic sensor was obtained under different magnetic field intensities and bias conditions. The principal parameters used in the three-dimensional TCAD simulation are summarized in Table 1.

**Figure 3:**
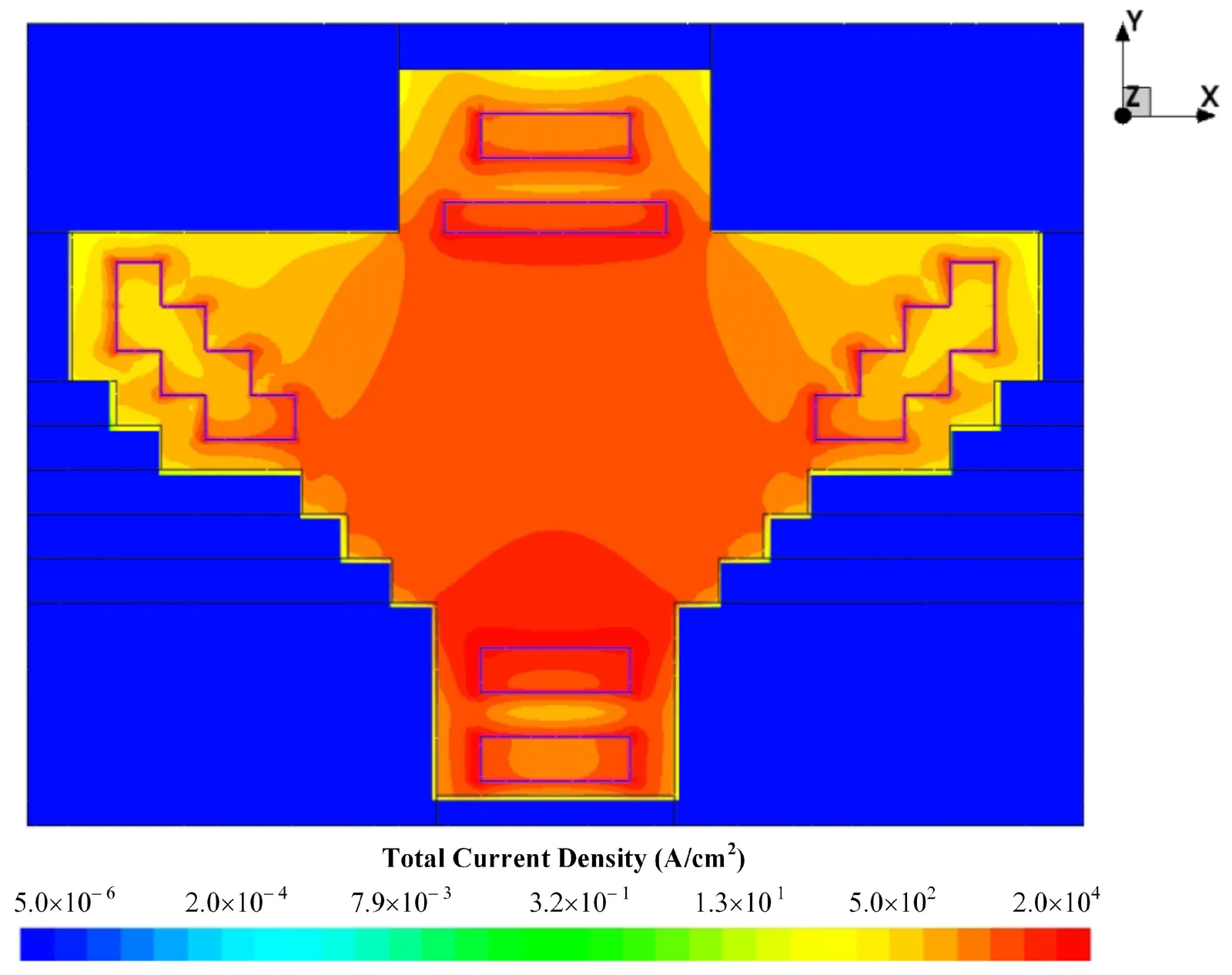
Simulated current density distributions of the μMFS without magnetic field.

**Figure 4:**
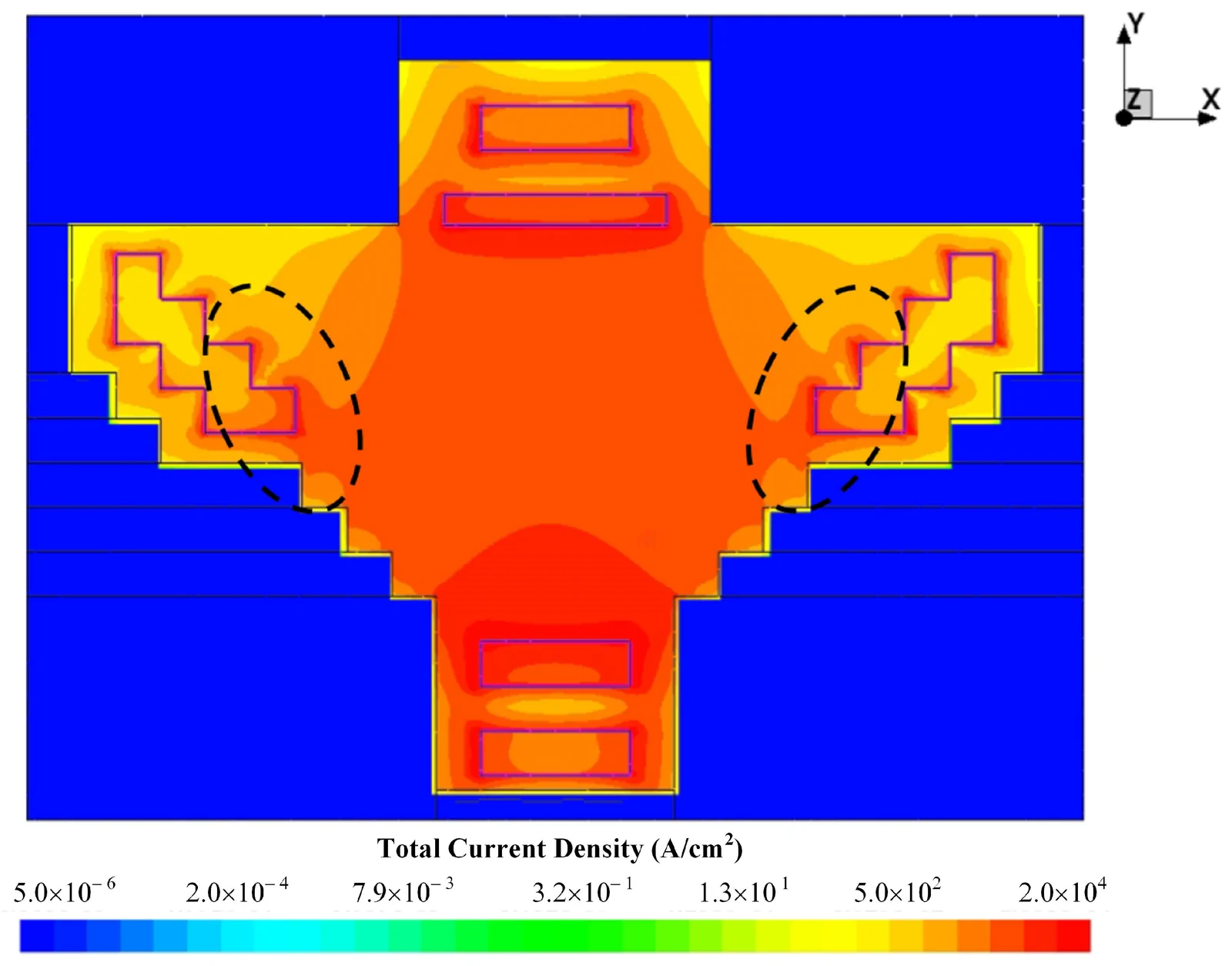
Simulated current density distributions of the μMFS under magnetic field of 100.

**Figure 5:**
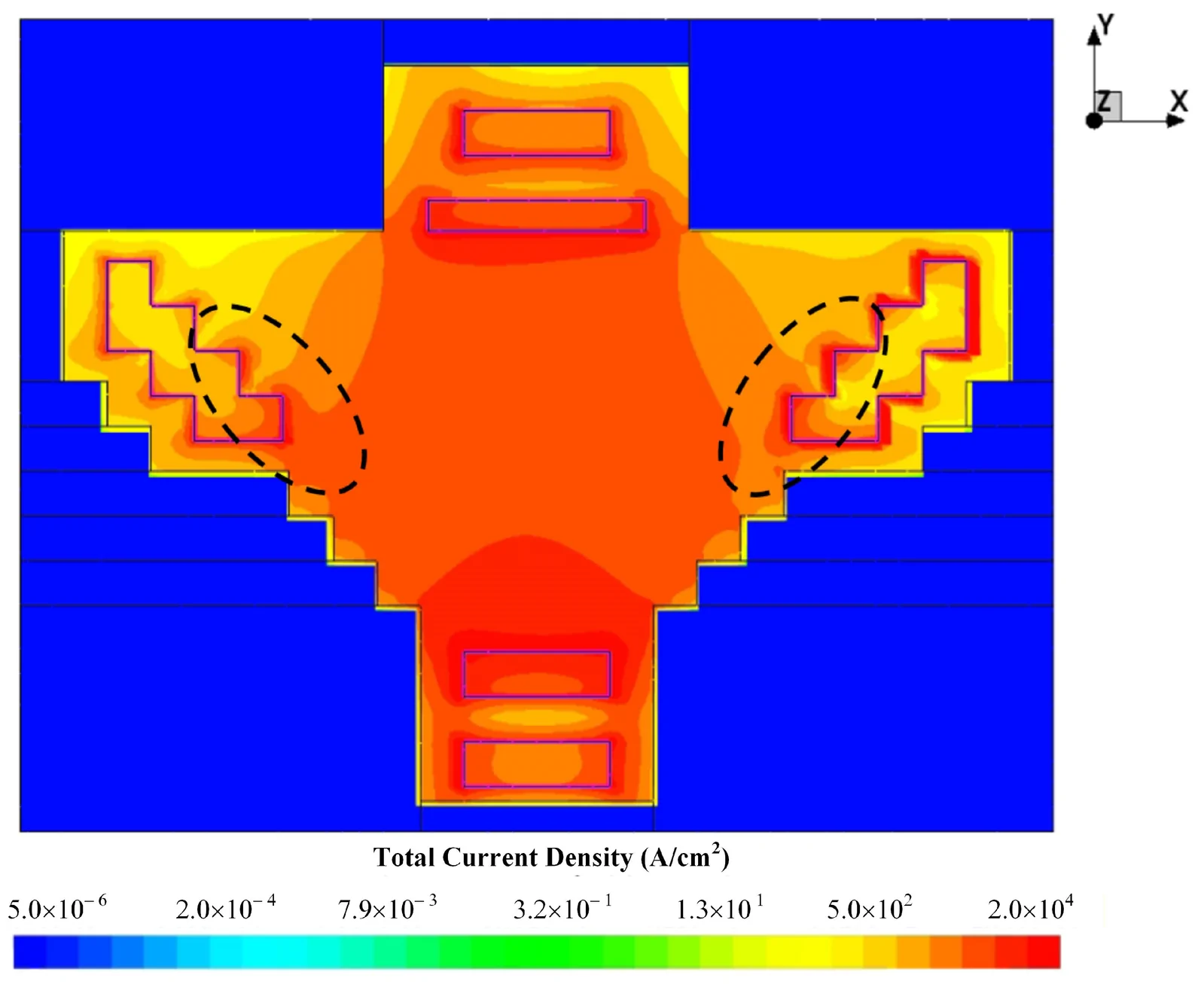
Simulated current density distributions of the μMFS under magnetic field of 300 mT.

Figure 3, Figure 4 and Figure 5 present the simulated current density distributions of the μMFS under magnetic fields of 0, 100, and 300 mT, respectively. The simulations were performed using TCAD to investigate the carrier transport behavior and to clarify the physical mechanism responsible for magnetic field sensing. The color contours represent the magnitude of the current density within the sensing element, allowing the carrier transport path and its variation under different magnetic field conditions to be directly visualized.

As shown in Figure 3, the current density is symmetrically distributed throughout the sensing element when no external magnetic field is applied. The carrier transport paths on the left and right sides exhibit nearly identical current density distributions, indicating that the injected carriers flow uniformly toward both collectors. Under this condition, no Lorentz force acts on the moving carriers, and therefore no lateral carrier deflection occurs. Consequently, the two collectors generate identical electrical potentials, resulting in a zero differential output voltage. In addition, the stepped collector geometry provides a larger carrier collection region, while the stepped STI confines the carrier transport within the active sensing region. This isolation structure effectively suppresses carrier leakage into the surrounding substrate and guides most carriers toward the collectors, thereby improving carrier collection efficiency and enhancing the electrical stability of the device.

When a perpendicular magnetic field of 100 mT is introduced, as illustrated in Figure 4, the moving carriers begin to experience the Lorentz force, causing the carrier transport path to deviate toward the left-hand side of the sensing element. Consequently, the current density enclosed by the left dashed circle becomes slightly higher than that on the opposite side. This asymmetric current distribution increases the carrier concentration collected by the left collector while reducing that of the right collector, thereby producing a measurable potential difference between the two sensing electrodes. The simulation confirms that the magnetic field is successfully converted into a differential electrical signal through carrier redistribution inside the magnetic transistor.

The carrier deflection becomes more pronounced when the magnetic field increases to 300 mT, as shown in Figure 5. Compared with Figure 4, a substantially larger current density is observed in the left highlighted region, whereas the current density near the right collector decreases accordingly. The enhanced carrier accumulation indicates that a stronger Lorentz force produces a greater lateral displacement of the injected carriers, resulting in a larger imbalance between the two collectors. Consequently, the differential output voltage increases with increasing magnetic field intensity. These simulation results demonstrate that the sensing mechanism of the μMFS originates from the magnetic-field-induced redistribution of carrier transport. Furthermore, the stepped collector geometry and STI isolation effectively confine the carrier flow within the active region, enabling the Lorentz-force-induced carrier deflection to be converted into a larger differential output signal.

**Figure 6:**
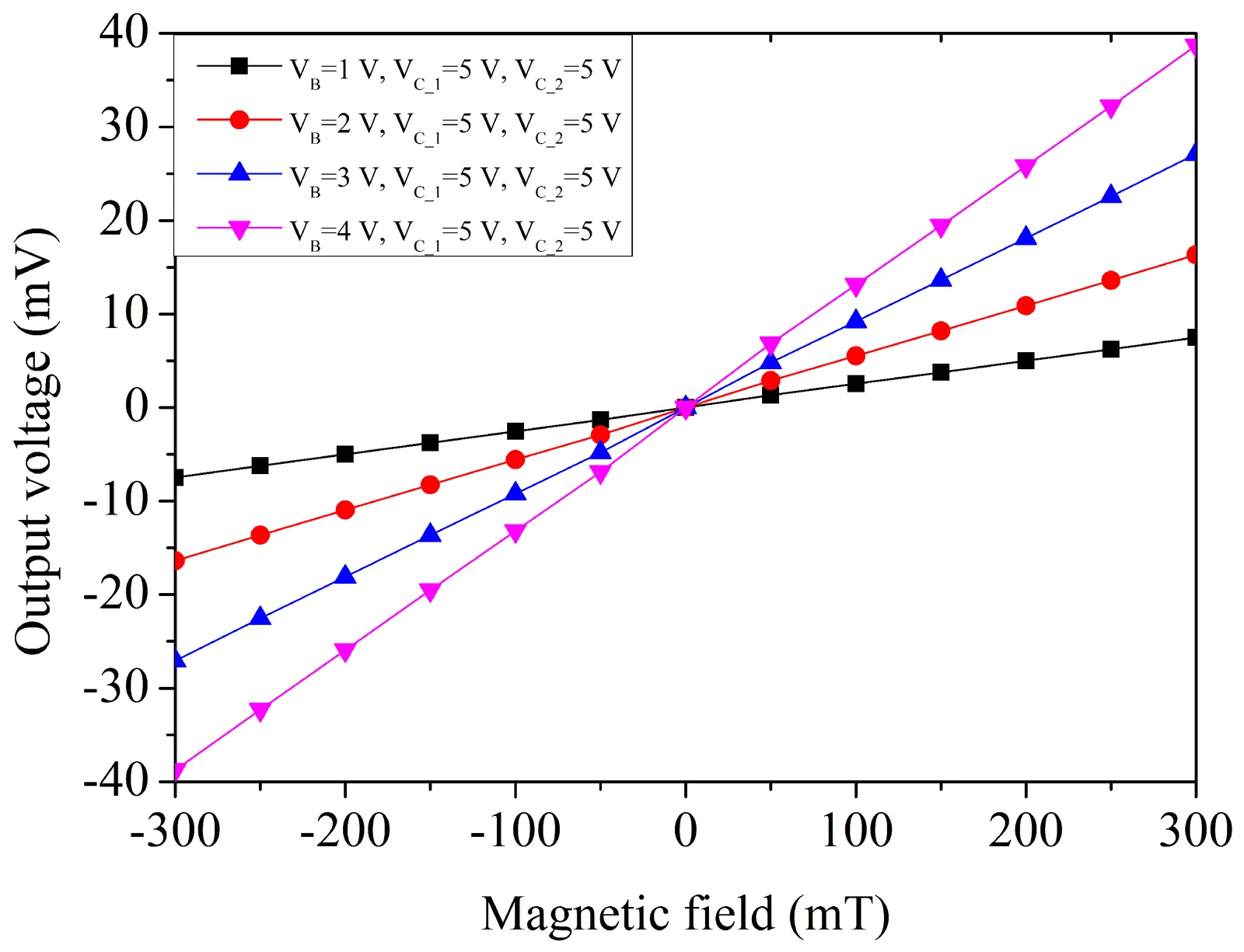
Simulated output characteristics of the μMFS under.

Figure 6 presents the simulated output characteristics of the μMFS under different base bias voltages, where V_B_ denotes the base bias voltage, V_C_1_ is the collector bias voltage and V_C_2_ is the additional collector bias voltage. During the simulation, the collectors and the additional collectors were biased at 5 V. The applied magnetic field was swept from −300 to 300 mT to evaluate the magnetic response over the entire operating range. As shown in Figure 6, the output voltage exhibits an excellent linear relationship with the applied magnetic field for all base bias conditions. The nearly symmetric responses with respect to zero magnetic field indicate that the sensor maintains stable differential operation over both positive and negative magnetic fields. Furthermore, the slope of the output voltage curve increases continuously as the base bias voltage is increased from 1 to 4 V, demonstrating a significant enhancement in magnetic sensitivity. At a magnetic field of 300 mT, the simulated output voltage increases from approximately 7.5 mV at V_B_ = 1 V to approximately 38.5 mV at V_B_ = 4 V, corresponding to an increase of more than five times in output magnitude.

The simulated sensitivities are approximately 25, 53, 90, and 128 mV/T for base bias voltages of 1, 2, 3, and 4 V, respectively. These results clearly indicate that increasing the base bias substantially improves the magnetic sensitivity while preserving excellent linearity throughout the measurement range. In addition, the output curves become progressively steeper with increasing base bias, indicating that the sensor exhibits a higher voltage response to the same magnetic-field variation. The simulation therefore confirms that the base bias voltage is an effective operating parameter for enhancing the output response of the μMFS without degrading its linear sensing characteristics.

## 3. Fabrication of the Micro Magnetic Field Sensor

**Figure 7:**
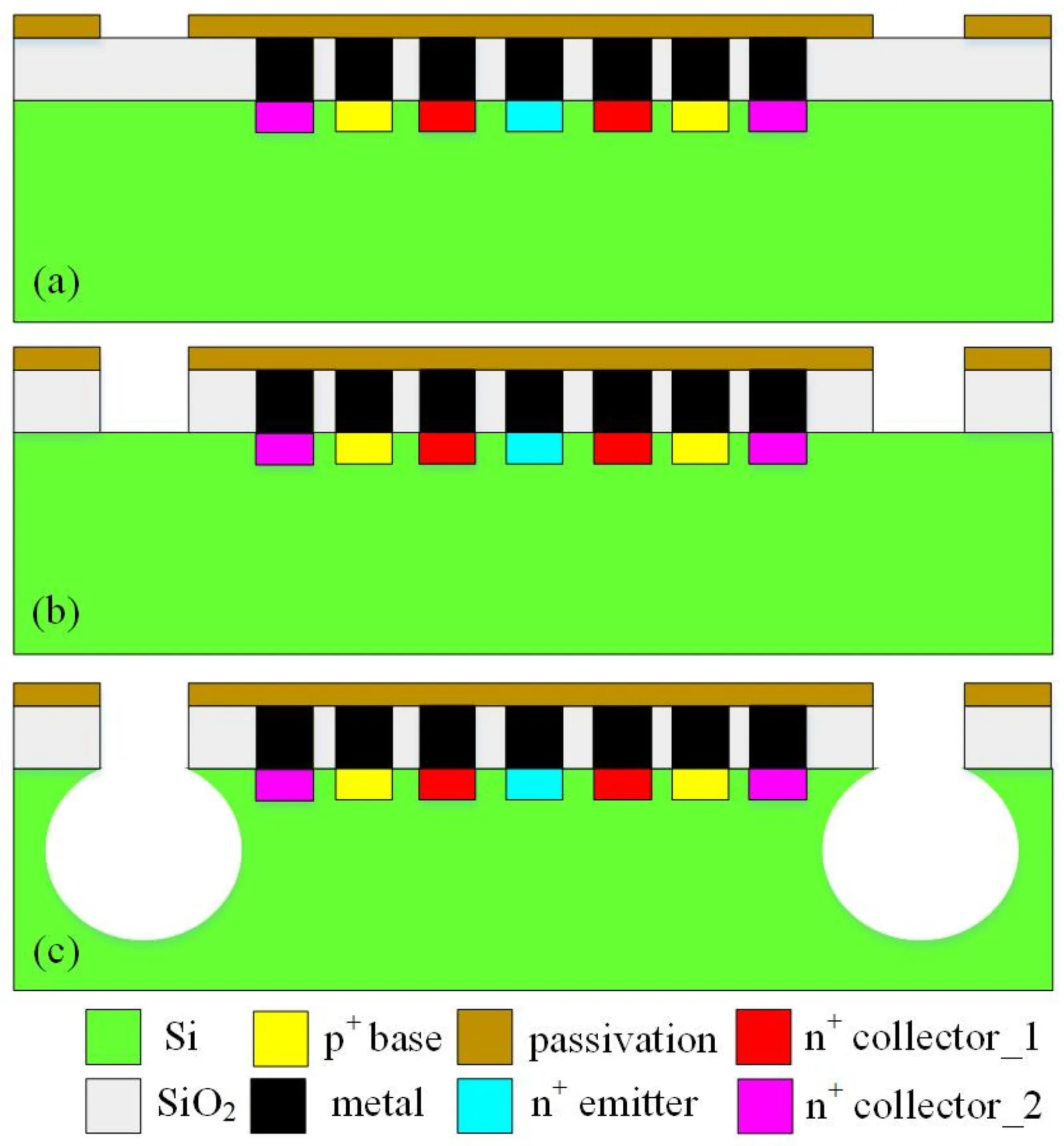
Fabrication flow of the μMFS: (**a**) after the CMOS process; (**b**) etching SiO_2_; (**c**) removing silicon substrate.

Figure 7 illustrates the fabrication procedure of the μMFS, which combines a standard CMOS process with a simple post-CMOS micromachining technique. This approach enables the sensor to be fabricated using a commercial CMOS foundry without introducing any additional process modifications, thereby providing excellent compatibility with conventional integrated circuit technology while maintaining a low fabrication cost. As shown in Figure 7a, the magnetic sensing element is first fabricated using the standard Taiwan Semiconductor Manufacturing Company (TSMC) 0.35 μm CMOS process. The sensing structure consists of a p-type silicon substrate, n-type emitter, p-type base, primary collectors, additional collectors, metal interconnections, silicon dioxide insulation layers, and the passivation layer. After completion of the CMOS fabrication, all active regions and electrical interconnections are fully integrated within the silicon substrate, providing the fundamental structure of the magnetic sensor.

The post-CMOS micromachining process begins with the removal of the silicon dioxide layer surrounding the sensing element, as illustrated in Figure 7b. Reactive ion etching (RIE) with CF_4_ plasma is employed to anisotropically etch the exposed silicon dioxide while preserving the underlying silicon and metal structures. This step opens the etching windows required for the subsequent substrate micromachining process and accurately defines the regions where silicon removal will be performed. Following the oxide etching process, the exposed silicon substrate is selectively etched using SF_6_-based reactive ion etching, as shown in Figure 7c. The isotropic silicon etching forms air cavities beneath both sides of the sensing element, effectively removing the surrounding silicon substrate without affecting the active sensing region. The introduction of these cavities significantly suppresses substrate leakage current and weakens electrical coupling between the sensing element and the bulk silicon. Consequently, parasitic current paths are reduced, allowing a larger proportion of the injected carriers to participate in the magnetic sensing process. The combination of CMOS fabrication and post-CMOS substrate micromachining therefore provides an effective approach for improving the electrical isolation and sensing performance of the μMFS while preserving full CMOS compatibility.

**Figure 8:**
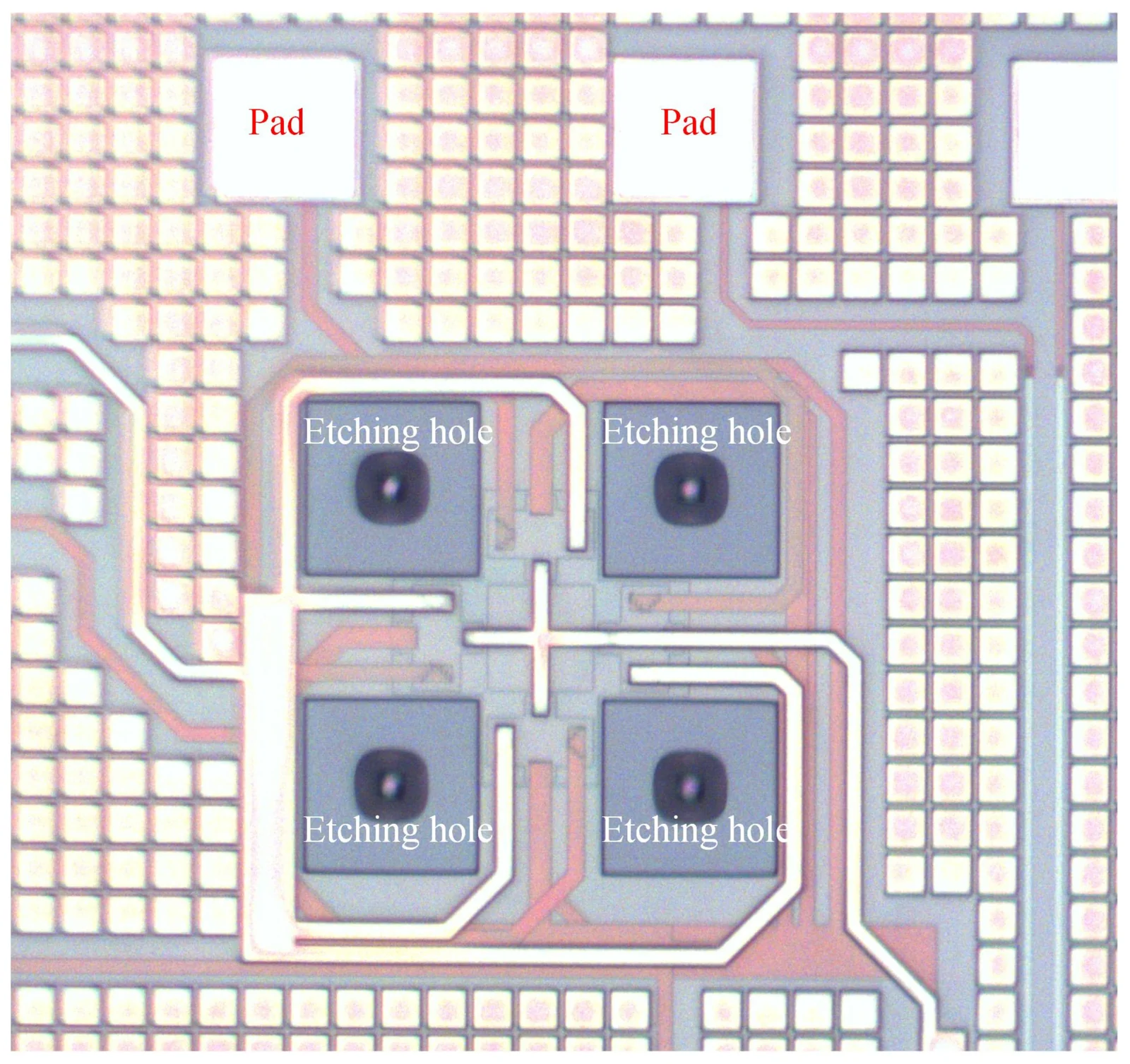
Optical image of the μMFS.

**Figure 9:**
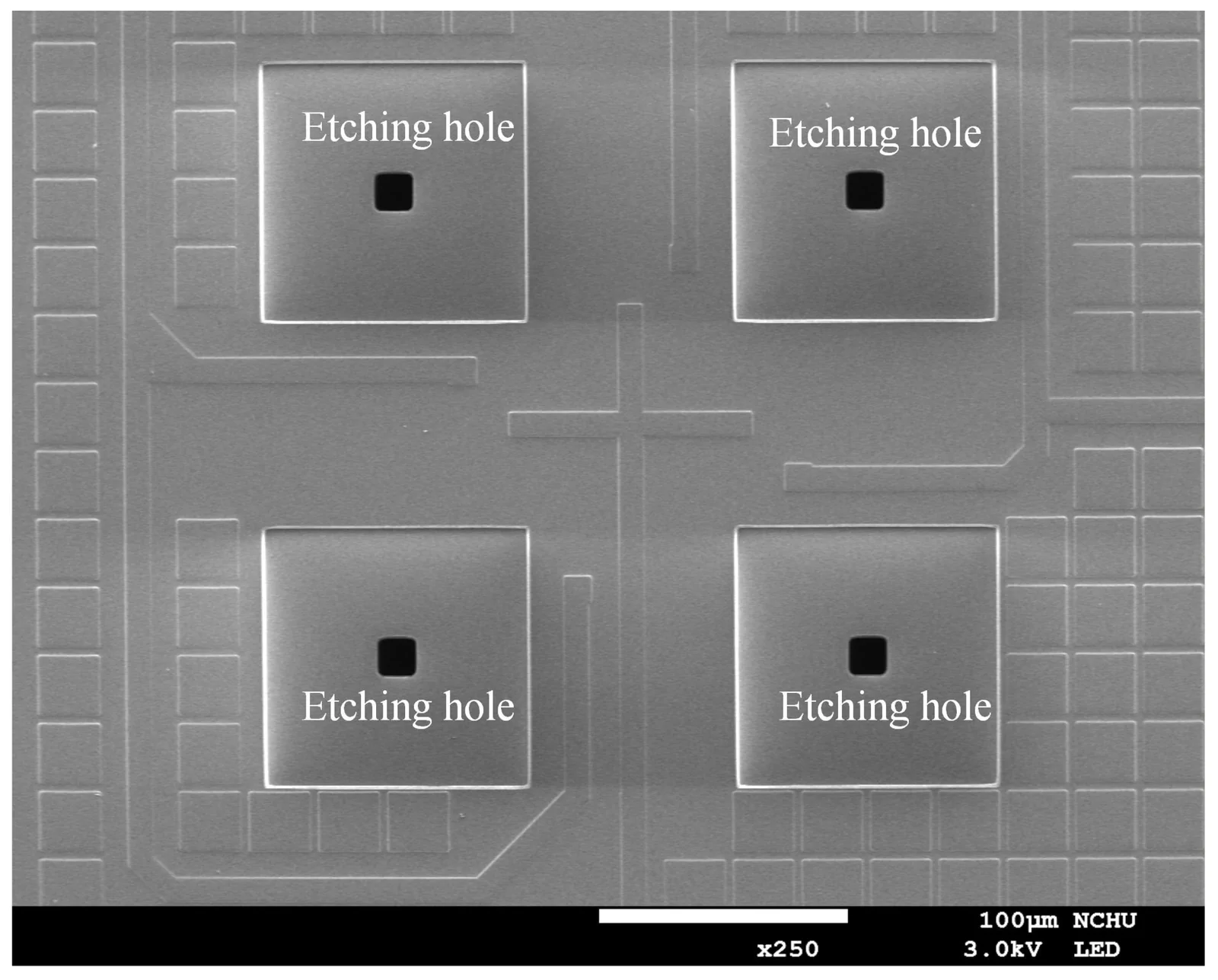
Scanning electron microscopy (SEM) of the μMFS.

**Figure 10:**
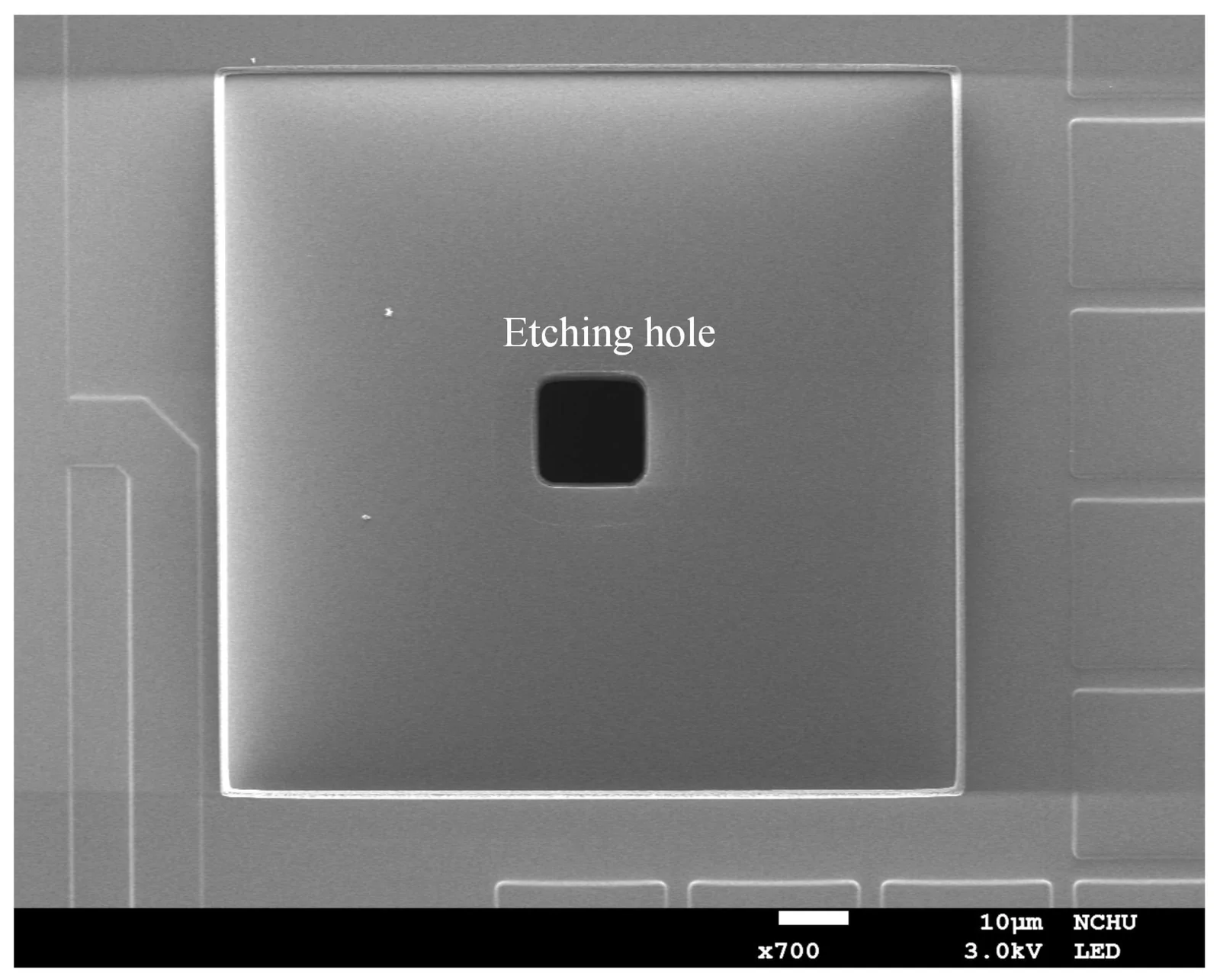
SEM image of the etching hole.

**Figure 11:**
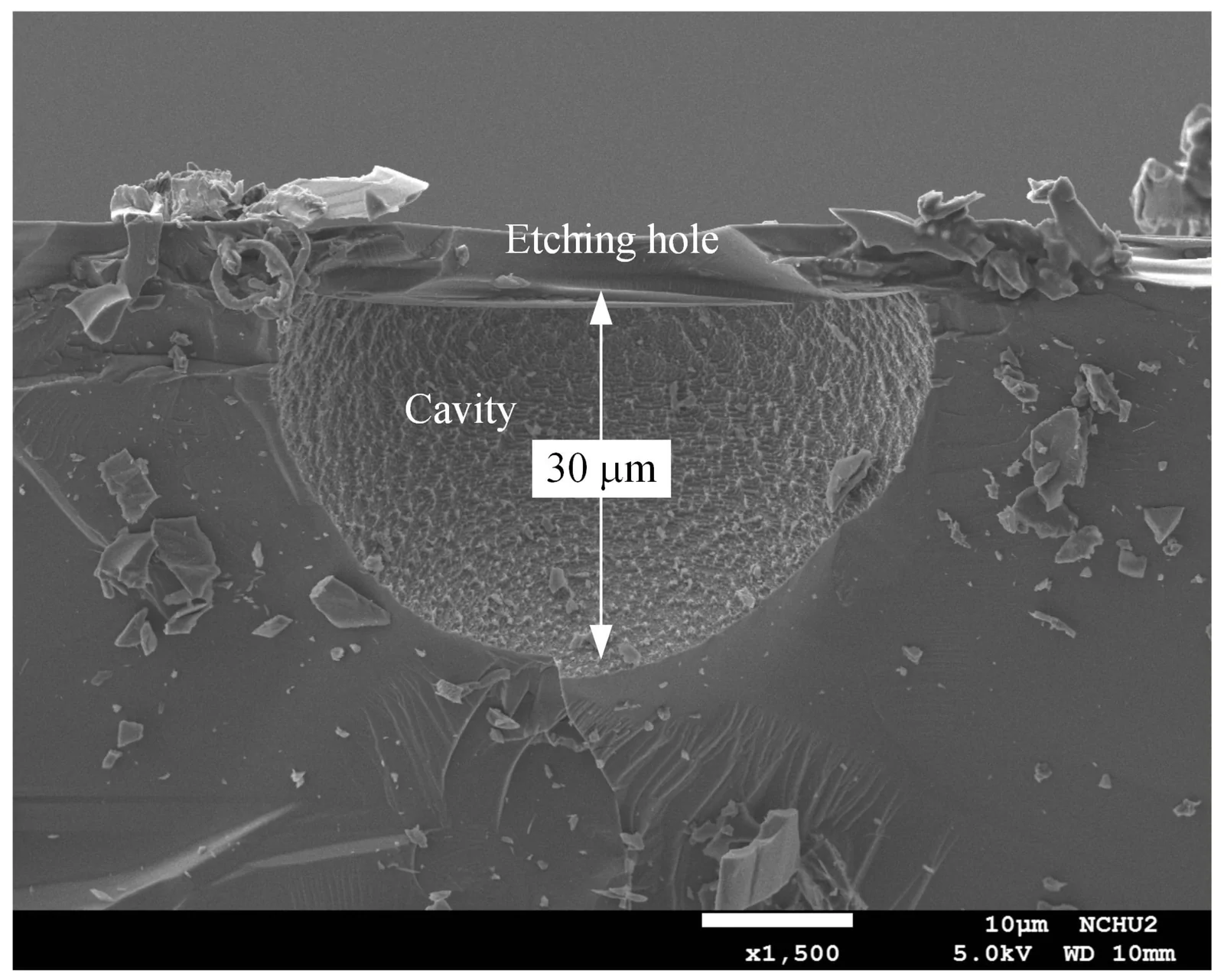
Cross-sectional SEM image of the cavity beneath the etching hole.

Figure 8, Figure 9, Figure 10 and Figure 11 present the fabricated μMFS after completion of the post-CMOS micromachining process. The optical micrograph shown in Figure 8 confirms that the fabricated device is consistent with the designed layout. Four identical magnetic sensing elements are symmetrically arranged in a cross-shaped configuration, and the electrical interconnection lines remain intact after the post-processing steps. In addition, each sensing element contains an individual etching hole located at the center of the cavity region. These etching holes serve as the access openings for the subsequent silicon substrate etching and are uniformly distributed among the four sensing elements, indicating excellent fabrication uniformity and alignment accuracy.

The overall surface morphology of the fabricated device is further examined using scanning electron microscopy (SEM), as shown in Figure 9. The SEM image demonstrates that the sensing elements retain their designed geometry after the post-CMOS process, and no noticeable structural deformation, fracture, or delamination is observed. The etching holes exhibit well-defined square profiles with smooth sidewalls, indicating that the reactive ion etching process provides good dimensional control and maintains the integrity of the surrounding passivation layer. The agreement between the optical image and the SEM observation confirms that the post-processing sequence does not introduce observable damage to the active sensing structure. A magnified SEM image of a single sensing element is presented in Figure 10. The etching hole is clearly formed at the center of the square etching window with a regular geometry and clean boundary, demonstrating precise lithographic pattern transfer and anisotropic oxide etching. The location of the etching hole is carefully designed to provide an effective pathway for the subsequent silicon substrate removal while minimizing its influence on the active magnetic sensing region. This design allows the silicon etchant to penetrate efficiently beneath the sensing structure and promotes the formation of an isolated cavity without affecting the electrical contacts or metal interconnections.

The cross-sectional SEM image shown in Figure 11 verifies the successful formation of the cavity beneath the etching hole. A cavity with a depth of approximately 30 μm is observed beneath the sensing element, confirming that the SF_6_-based reactive ion etching process effectively removes the underlying silicon substrate. The cavity is well confined beneath the sensing region and exhibits a smooth isotropic etching profile, indicating a stable and controllable post-CMOS micromachining process. The formation of the cavity significantly reduces the volume of bulk silicon surrounding the sensing element, thereby suppressing substrate leakage current and weakening parasitic electrical coupling through the silicon substrate. Together with the STI isolation structure, the suspended cavity further enhances electrical isolation and improves carrier confinement within the active sensing region. These fabrication results demonstrate that the post-CMOS process can successfully realize the designed μMFS.

## 4. Results

**Figure 12:**
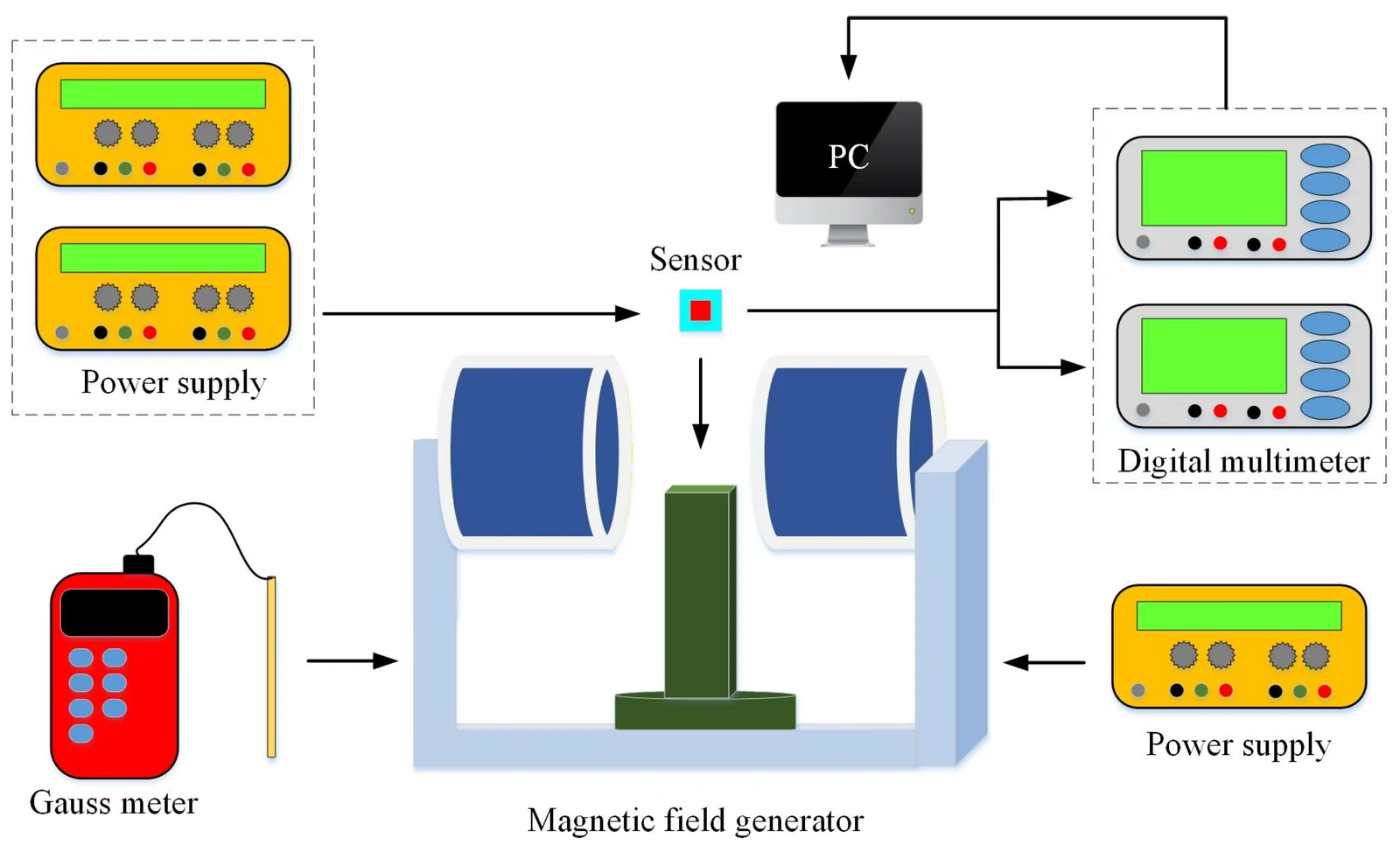
Experimental setup for the μMFS.

Figure 12 illustrates the experimental setup used to characterize the μMFS. The measurement system consists of a magnetic field generator, power supplies, digital multimeters, a Gauss meter, a sensor fixture, and a personal computer (PC). The μMFS is mounted on the fixture and positioned at the center of the magnetic field generator to ensure that a uniform magnetic field is applied perpendicular to the sensing surface during the measurements. Separate DC power supplies are employed to provide the required operating voltages for both the μMFS and the magnetic field generator. The magnetic field intensity is adjusted by varying the driving voltage supplied to the magnetic field generator. To ensure measurement accuracy, the generated magnetic field is calibrated using a Gauss meter before each measurement. The calibrated magnetic field serves as the reference value for evaluating the electrical response of the sensor. The output signals of the μMFS are measured using two digital multimeters, which simultaneously monitor the output voltages of the differential sensing electrodes. The measured data are transferred to a personal computer for data acquisition, storage, and subsequent analysis. During the characterization, the magnetic field is gradually varied over the desired measurement range while maintaining fixed bias voltages on the sensor. The corresponding output voltages are continuously recorded to establish the relationship between the applied magnetic field and the sensor response. Based on these measured results, the output characteristics and magnetic sensitivity of the μMFS are subsequently evaluated. During the measurements, a 1 kΩ external load resistor was connected to each collector output to convert the output current into the output voltage. The total operating current of the sensor was approximately 5 mA under the measurement conditions, corresponding to a power consumption of approximately 25 mW with a 5 V supply.

**Figure 13:**
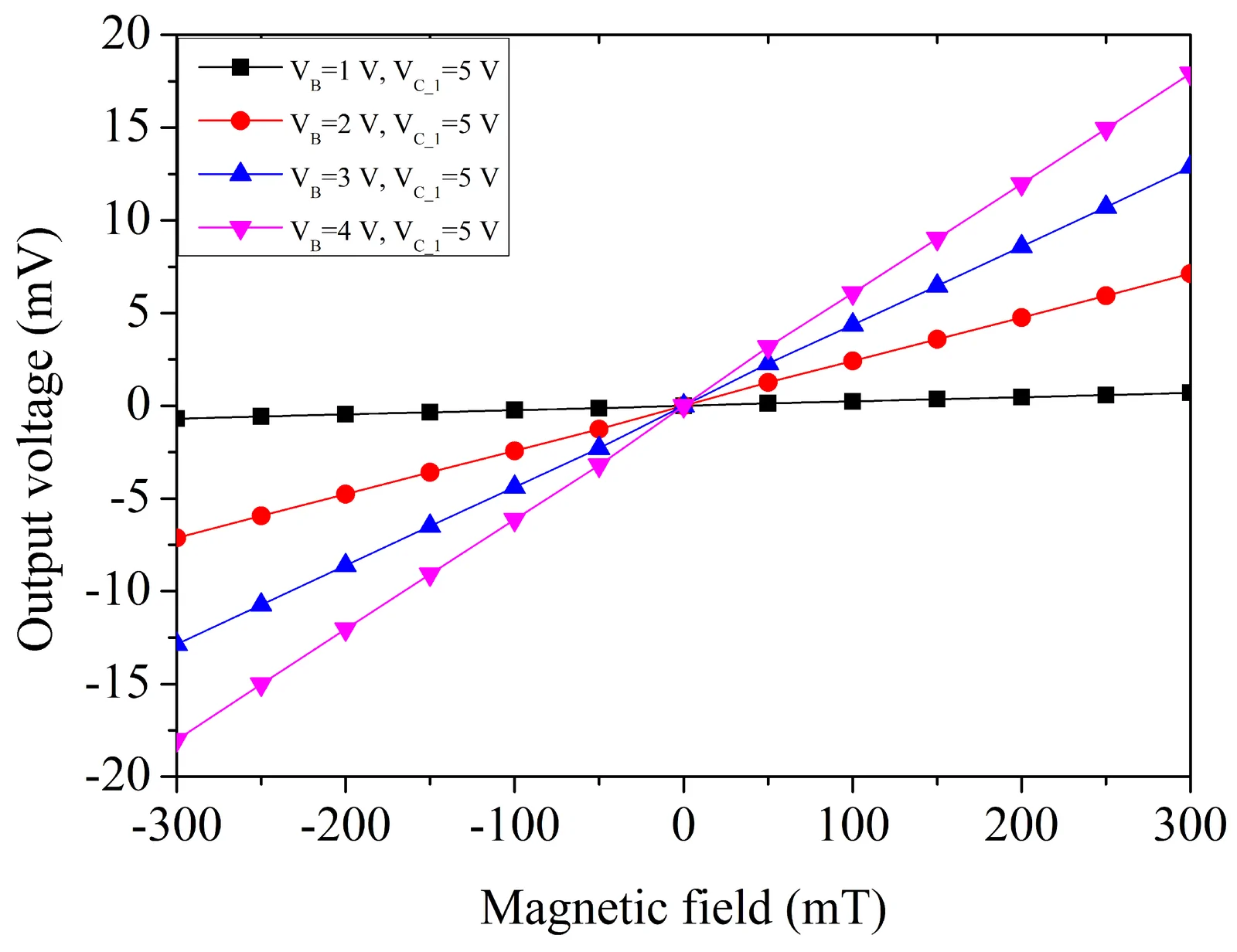
Measured output characteristics of the μMFS without additional collector bias.

Figure 13 shows the measured output characteristics of the μMFS under different base bias voltages, where V_B_ denotes the base bias voltage and V_C_1_ is the collector bias voltage. During the measurements, the collectors were biased at 5 V, while the additional collectors were left floating. The applied magnetic field was swept from −300 to 300 mT to evaluate the sensor performance under different operating conditions. As shown in Figure 13, the output voltage varies almost linearly with the applied magnetic field over the entire measurement range. For each operating condition, the output voltage increases gradually as the magnetic field changes from negative to positive values, exhibiting a nearly symmetric response with respect to zero magnetic field. The linear characteristics indicate that the fabricated sensor provides a stable voltage response and is capable of accurately detecting variations in magnetic field intensity. The effect of the base bias voltage on the sensor performance is also clearly observed. As the base bias voltage increases from 1 to 4 V, the slope of the output-voltage curves becomes progressively steeper, indicating a significant improvement in magnetic sensitivity. At an applied magnetic field of 300 mT, the measured output voltage increases from approximately 0.6 mV at V_B_ = 1 V to approximately 18 mV at V_B_ = 4 V. The sensitivity is 60 mV/T for base bias voltages of 4 V.

**Figure 14:**
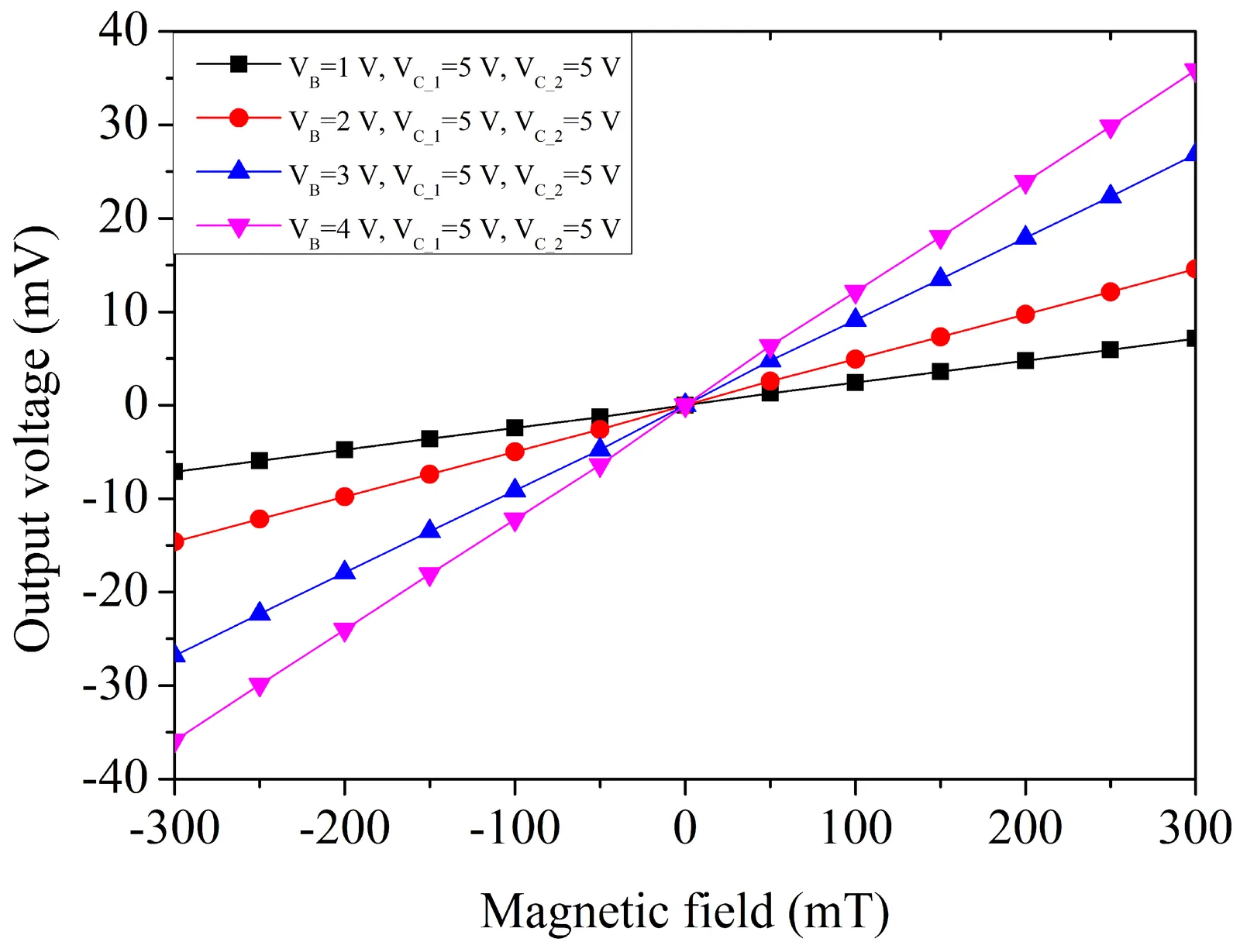
Measured output characteristics of the μMFS with additional collector bias.

Figure 14 presents the measured output characteristics of the μMFS, where V_B_ denotes the base bias voltage, V_C_1_ is the collector bias voltage and V_C_2_ is the additional collector bias voltage. All collectors and additional collectors were biased at 5 V. The base bias voltage was varied from 1 to 4 V, while the external magnetic field was swept from −300 to 300 mT to evaluate the influence of the additional collector bias on the sensor performance. As shown in Figure 14, the output voltage exhibits an excellent linear relationship with the applied magnetic field over the entire measurement range for all base bias voltages. Similar to the results presented in Figure 13, the output voltage increases monotonically with increasing magnetic field, and the slope of each response curve becomes progressively steeper as the base bias voltage increases. The corresponding measured sensitivities are 120 mV/T for base bias voltages of 4 V. These results indicate that the μMFS maintains excellent linearity while providing significantly enhanced magnetic sensitivity at higher base bias voltages.

It should be noted that the structural contribution and the biasing effect are different. The additional collector is a structural feature of the sensor, whereas the results presented in Figure 13 and Figure 14 evaluate the influence of applying a bias voltage to the additional collector. Therefore, the comparison demonstrates the effect of the additional collector bias on the sensor performance rather than a comparison between devices with and without the additional collector. A comparison between Figure 13 and Figure 14 clearly demonstrates the effect of applying a bias voltage to the additional collector. Under identical base bias conditions, the output voltage in Figure 14 is substantially higher than that in Figure 13 throughout the entire magnetic-field range. For example, at a magnetic field of 300 mT and a base bias of 4 V, the measured output voltage increases from approximately 18 mV in Figure 13 to approximately 36 mV in Figure 14, representing nearly a twofold enhancement. This improvement indicates that the biased additional collector effectively strengthens carrier collection and increases the differential output voltage, thereby significantly improving the magnetic sensitivity without sacrificing the linear characteristics of the sensor. The experimental results are also consistent with the TCAD simulation presented in Figure 6. Both the simulation and measurement show that increasing the base bias enhances the output response while preserving an approximately linear relationship between the output voltage and the applied magnetic field. Although the measured maximum sensitivity (120 mV/T) is slightly lower than the simulated value (128 mV/T), the deviation is approximately 6.3%, indicating good agreement between the simulation and experimental results. The discrepancy is mainly attributed to practical factors that are difficult to fully incorporate into the TCAD simulation, including fabrication tolerances, contact resistance, process variations, carrier recombination, and measurement noise. Overall, the close agreement verifies the validity of the sensor design and the effectiveness of the simulation model.

Table 2 compares the sensitivity of the proposed *z*-axis μMFS with those reported for previously published *z*-axis μMFSs. As summarized in Table 2, the reported sensitivities range from 19.2 to 119 mV/T, including 19.2 mV/T for Dai et al. [9], 27.8 mV/T for Chen et al. [27], 119 mV/T for Wu et al. [28], 77.4 mV/T for Zhao et al. [29], and 30 mV/T for Sileo et al. [30]. The proposed μMFS achieves a measured sensitivity of 120 mV/T, which is comparable to the highest sensitivity reported for CMOS-compatible *z*-axis magnetic field sensors. The improved sensitivity is attributed to the incorporation of an additional collector, together with the four-element cross-shaped configuration and the STI isolation structure, which enhance carrier transport, increase carrier collection efficiency, and suppress leakage current. These results demonstrate that the proposed sensor provides competitive magnetic sensing performance while maintaining compatibility with the standard CMOS process and a simple post-CMOS fabrication procedure.

## 5. Conclusions

A CMOS-compatible *z*-axis μMFS incorporating an additional collector has been successfully designed, fabricated, and characterized in this study. The sensor consists of four identical magnetic sensing elements arranged in a cross-shaped configuration, which effectively suppresses electrical interference among individual sensing elements while improving measurement stability. In addition, the combination of STI and a post-CMOS micromachining process forms isolated cavities beneath the sensing elements, thereby reducing substrate leakage current and enhancing electrical isolation. The sensing characteristics were first investigated using three-dimensional TCAD simulations. The simulation results demonstrated that the carrier transport was gradually deflected by the Lorentz force as the applied magnetic field increased, leading to a redistribution of the collector current and the generation of a differential output voltage. Furthermore, the simulation confirmed that increasing the base bias voltage significantly enhanced the magnetic sensitivity while maintaining excellent linearity over the investigated magnetic-field range. The μMFS was fabricated using a commercial CMOS process followed by a simple post-CMOS dry-etching process employing CF_4_ and SF_6_ reactive ion etching. Optical microscope and SEM observations verified that the fabricated structures agreed well with the designed layout, and a cavity with a depth of approximately 30 μm was successfully formed beneath each sensing element. Experimental measurements demonstrated that the sensor exhibited a linear output response over the magnetic-field range from −300 to 300 mT. When the collectors and the additional collectors were biased at 5 V and the base bias was 4 V, the sensor achieved a maximum measured sensitivity of 120 mV/T, which is higher than those reported for previously published μMFSs. The μMFS demonstrates high sensitivity, good linearity, and full compatibility with the standard CMOS-MEMS process, indicating its potential for integrated magnetic sensing applications.

## Figures and Tables

**Table 1:** Parameters used for the TCAD simulation.

Parameter	Value
Simulation tool	Synopsys Sentaurus TCAD
Simulation type	Three-dimensional
CMOS technology	TSMC 0.35 μm CMOS
Substrate	P-type silicon
Magnetic field	−300 to 300 mT
Base bias (V_B_ )	1–4 V
Collector bias (V__C1_ )	5 V
Additional collector bias (V__C2_)	5 V

**Table 2:** Sensitivity of various *z*-axis magnetic field sensors.

Authors	Sensitivity (mV/T)
Dai et al. [9]	19.2
Chen et al. [27]	27.8
Wu et al. [28]	119
Zhao et al. [29]	77.4
Sileo et al. [30]	30
This work	120

## Data Availability

The data presented in this study are available on request from the corresponding author. The data are not publicly available due to ongoing research activities.
